# Pharmacodynamic analysis and concentration mapping for efficient delivery through the FUS-induced BBB opening in non-human primates i*n vivo*

**DOI:** 10.1186/2050-5736-3-S1-P33

**Published:** 2015-06-30

**Authors:** Gesthimani Samiotaki, Maria Eleni (Marilena) Karakatsani, Shih-Ying Wu, Amanda Marie Buch, Elisa Konofagou

**Affiliations:** 1 Columbia University, New York, New York, United States

## Background/introduction

FUS in conjunction with systemically administered microbubbles has been previously shown to open the Blood-Brain Barrier (BBB) locally, non-invasively and reversibly in non-human primates. However, a trans-BBB pharmacodynamic analysis has not been performed as of yet. The objective of this study was to perform such an analysis, i.e. permeability, relaxivity and gadolinium concentration mapping, of the NHP brain *in vivo* in order to further investigate the effect of FUS, and its dependence on the acoustic parameters used for safe and efficient drug-delivery as well as the gray *vs*. white matter occurrence.

## Methods

Two brain structures, the caudate and the putamen, were targeted in three rhesus macaques using FUS (center frequency: 500 kHz; pulse length: 5,000 cycles; PRF: 2 Hz; sonication duration: 120 s; peak negative pressure: 300-500 kPa) immediately after the IV administration of monodisperse bubbles (diameter: 4-5 μm, 2.5*108#/kg). Following sonication, the macaques were placed in a 3T MR scanner (Philips Medical System, Andover, MA, USA). Five pre-contrast 3D Spoiled Gradient Echo (SPGR) images (TR/TE: 10/4ms, FA: 5o-35o, NEX: 3, matrix: 256x256, resolution: 1x1x1 mm3) were acquired and used for variable flip angle *VFA) based T1 relaxivity mapping. Subsequently, Dynamic Contrast Enhanced (DCE) imaging was performed, with the acquisition of 90 dynamic T1-weighted 3D repetitions (TR/TE: 4.2/1.7 ms; matrix: 256x256, resolution 1x1x2 mm3). The data of the DCE were processed off-line using a customized Matlab-based algorithm and fitted to the General Kinetic Model using the Patlak method to generate permeability maps.

Quantitative permeability changes (Ktrans) and the volume of BBB opening after excluding the vasculature were obtained based on the T1 relaxivity maps. The areas of gray and white matter where BBB opening was induced were also determined, since they have distinctive characteristic relaxivity times. Gadolinium concentration [Gd] maps were then calculated from the T1, pre map before MR-CA injection and the T1, post maps after MR-CA injection using the following equation: [Gd] =1/r_Gd (1/T_(1, post) -1/T_(1, pre)). Vasculature and CSF were excluded from the opening volume based on their T1 relaxivity which was measured to be above 1400 ms.

## Results and conclusions

The volume of opening increased from 92±10 mm3 to 262±34 mm3 with a pressure increase from 300 kPa to 500 kPa increased. The average permeability was increased from 1.0531 ± 0.0761*10-4 s-1 to 1.863 ± 0.132*10-4 s-1 with the same pressure increase. When targeting the putamen, an average of 95% of the BBB opening regions was contained in the grey matter (T1 relaxivity range: 1001-1400 ms), while when targeting the caudate 87% of the BBB-opened regions were in the gray matter and the rest in the white (T1 relaxivity range: 600-1000 ms). No edema or hemorrhage was detected in any of the cases studied. FUS-induced drug delivery efficiency was measured *in vivo* for the first time and increased with the acoustic pressure used; the amount of gadolinium in the opened BBB area increased from 10 μg to 20 μg on average when pressure increased from 300 to 600 kPa. This type of analysis as performed in this study may prove critical for clinical applications.

**Figure 1:**
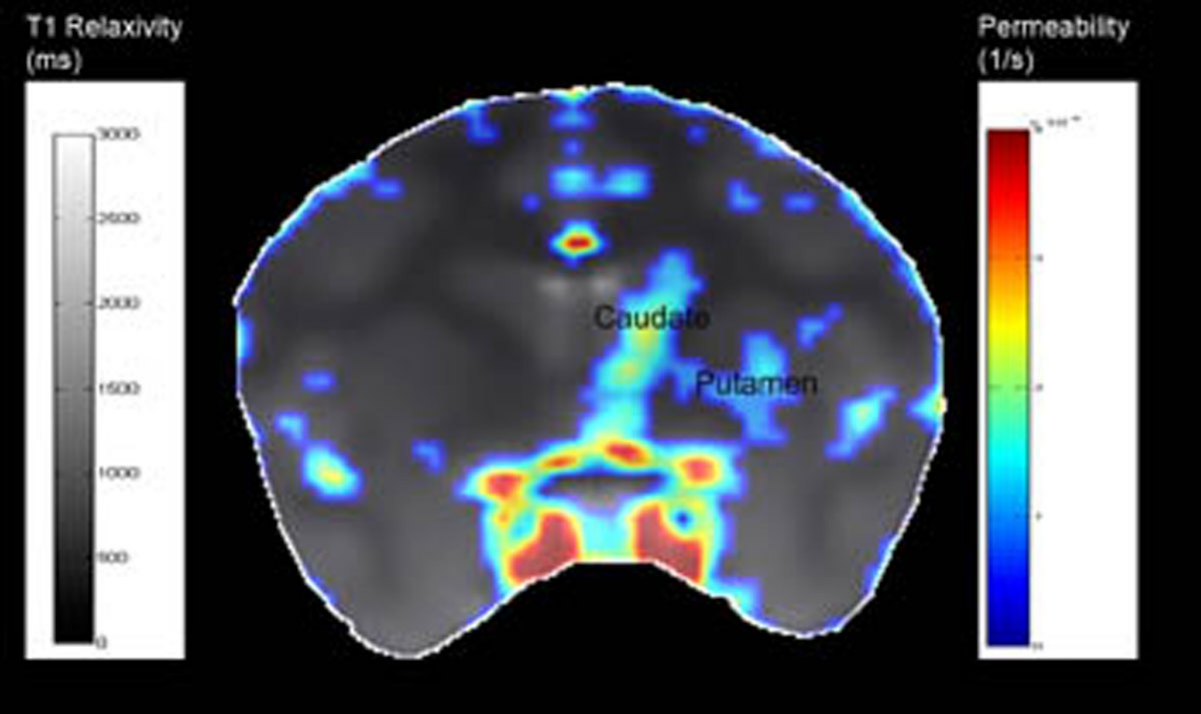
T1 map overlaid with permeability map in a coronal view

